# The Effect of Joining the ASSH Hand Trauma Center Network on the Volume and Severity of Pediatric Hand Trauma Transfers

**DOI:** 10.1016/j.jhsg.2023.06.011

**Published:** 2023-07-22

**Authors:** Ebubechi Adindu, Bryan Head, Bryce Bell

**Affiliations:** ∗ Baylor College of Medicine, Houston, TX; † Department of Orthopedic Surgery, Texas Children's Hospital, Houston, TX

**Keywords:** Hand surgery, Hand trauma, Hand trauma transfers, Pediatric hand surgery

## Abstract

**Purpose:**

The purpose of our study was to evaluate how the volume and severity of pediatric hand trauma is affected after enrollment into the American Society for Surgery of the Hand Trauma Center Network.

**Methods:**

We performed a retrospective review using the patient database from our affiliated level-I pediatric trauma center. With this patient database, we compiled all emergent hand trauma transfers from February 2018 to January 2022. We compared the monthly volume, Injury Severity Score, and quarterly payor status between hand trauma transfer patients before and after enrollment into the Hand Trauma Center Network in February 2019.

**Results:**

The average number of monthly transfers increased after joining the Hand Trauma Center Network compared with the years after February 2019. Additionally, the percentage of patients using commercial insurance increased after joining the Hand Trauma Center Network when compared with that before February 2019. Lastly, the percentage of patients using Medicaid decreased after February 2019.

**Conclusions:**

Based on our findings, we believe that new institutions and providers can expect anywhere from a 10% to a 60% increase in hand trauma burden without a significant change in the severity of the trauma cases after joining the network.

**Type of study/level of evidence:**

Prognostic IV.

Hand injuries are among the most prevalent injuries in the United States. The 2019 National Hospital Ambulatory Medical Care Survey found that approximately 13% of all emergency center visits that year were because of injuries to the finger, hand, and/or wrist.^1^ Additionally, when compared with other types of injuries, hand injuries are more often associated with devastating long-term socioeconomic consequences. Annually, hand injuries account for more than $700 million of the total health care cost.^2^ Approximately 56% of that total cost is secondary to the decreased economic productivity that patients often face after injuring their hands and/or wrists.^2^ Therefore, hand injuries are not only common but also the most expensive type of injury in the United States.

When severe, hand injuries often require time-sensitive surgical treatment.^3^ However, despite how common and expensive hand injuries are, a striking lack of 24/7/365 hand surgical trauma call coverage exists in the United States.^4^, ^5^, ^6^ Additionally, barriers to timely access to care exist among patients who belong to racial minorities, are uninsured/underinsured, and/or live in rural areas.^7^, ^8^, ^9^ To address these discrepancies in the access to care for hand trauma emergencies, the American Society for Surgery of the Hand (ASSH) and American College of Surgeons (ACS) first discussed the idea of the Hand Trauma Center Network in 2007.^10^

Trauma centers included within the Hand Trauma Center Network do not need to be an ACS level-I trauma centers but must still be available for hand trauma 24/7 and perform revascularization and replantation procedures. Additionally, a center must have an on-call list of available hand surgeon attendings with or without trainees, and the director of the center must report hand trauma–related data.^10^

In a 2021 study, Eberlin et al^11^ assessed the trend of 24/7 hand-call coverage in the decade since work began on establishing the Hand Trauma Center Network. Eberlin et al^11^ found a minimal difference between surgeons who worked in facilities that offered 24/7 calls for hand trauma emergencies between 2010 (73%) and 2019 (70%). However, an increase was observed in the number of hand surgeons who opted against taking 24/7 calls between 2010 (18%) and 2019 (34%). This increase is, in part, due to the decreased number of hand surgeons working in areas with obligatory calls, 70% versus 50% in 2010 and 2019, respectively. Additionally, hand surgeons identified a disruption of elective surgical schedule and lifestyle inconvenience as other factors dissuading them from taking 24/7 trauma calls.^11^

Although many studies have addressed the availability of hand trauma calls and the current perspective on trauma calls among hand surgeons, a current knowledge gap exists on how enrollment in the Hand Trauma Network affects the volume and severity of hand trauma call. Therefore, the purpose of our study was to evaluate how the volume and severity of pediatric hand trauma is affected after enrollment into the Hand Trauma Center Network.

## Materials and Methods

We performed a retrospective review using the patient database from our affiliated level-I pediatric trauma center after obtaining approval from our institution’s ethical review board. With this patient database, we compiled all emergent hand trauma transfers from February 2018 to January 2022. Included in our compiled data were the patient Injury Severity Score (ISS), the reason for transfer, ICD 10 codes, and payor status at the time of transfer.

All patients included in this study were <18 years of age and were transferred secondary to injuries distal to the elbow including the nerve, tendon, open fractures, traumatic amputations, compartment syndrome, and dysvascularity of the hand or fingers. The primary end point of this study was to evaluate the volume and severity of trauma transfers before and after joining the ASSH Hand Trauma Center Network in February 2019. Our date range was selected to include transfers for the year before joining the network and the time since joining. Monthly trauma transfers, ISS, and quarterly patient payor status were compiled and compared between patients transferred before and after February 2019.

### Statistical method

A two-sample *t* test was performed to compare the monthly volume, ISS, and quarterly payor status between hand trauma transfer patients before and after February 2019. Confidence intervals (CIs) were calculated at 95%, and statistical significance was indicated by a *P* value of < .05.

## Results

A total of 430 hand trauma cases across 4 years were included in this study, 84 before joining the ASSH Hand Trauma Center Network and 346 afterward. As displayed in Table 1, an increase was observed in the average number of monthly transfers after joining the Hand Trauma Center Network compared with the years after February 2019 (7.00 vs 9.58, *P* < .01). This translates to an approximately 10% to 60% increase in patient volume based on the 95% CI (0.73–4.44, Table 1). No significant difference was noted between the average injury severity scores of hand trauma transfers before and after joining the Hand Trauma Center Network (3.05 vs 3.37, *P* = .56; Table 1).

**Table 1:** Average Number of Monthly Transfers and Severity Scores with “Before February 2019” as Reference

	Before February 2019	After February 2019	Mean Difference (95% CI)	*P* Value
Number of transfers	7	9.58	2.58 (0.73 to 4.44)	< .01∗
Injury Severity Scores	3.05	3.37	0.32 (–0.79 to 1.44)	.56

∗ *P* < .05

A significant increase was observed in the percentage of patients with commercial insurance after joining the Hand Trauma Center Network when compared with that before February 2019 (76% vs 68%, *P* = .04; Table 2). This was accompanied by a decrease (*P* < .05) in the percentage of patients with Medicaid insurance before and after February 2019 (18% vs 12%, *P* < .05). The percentage of patients who self-paid before and after joining the Hand Trauma Network remained approximately the same (9% vs 10%, *P* = .75).

**Table 2:** Quarterly % Payor Type with “Before February 2019” as Reference

Payor Type	% Before February 2019	% After February 2019	Mean Difference (95% CI)	*P* Value
Commercial insurance	68.2	75.98	7.78 (0.46 to 15.09)	.04∗
Medicaid	17.7	12.24	–5.5 (–10.87 to –0.04)	< .05∗
Self-pay	9.25	10.43	1.18 (–6.67 to 9.01)	.75

∗ *P* < .05.

## Discussion

The hand, fingers, and wrists are commonly injured in the pediatric patient population, with open fractures making up a significant percentage of the injuries.^12^, ^13^, ^14^ Many of these hand injuries require urgent surgical care that may not be readily available at local hospitals, leading to transfers to more advanced facilities with greater degrees of specialization.^15^^,^^16^ The ASSH Hand Trauma Center Network was established to improve the efficiency of this process and increase the availability of care for hand trauma.^10^ Therefore, knowledge of the effects on volume and severity of injuries after joining the Hand Trauma Center Network is a valuable set of parameters for hand surgeons working in eligible trauma centers.

Our institution noted a significant increase in the monthly volume of pediatric hand trauma cases after joining the Hand Trauma Center Network. Many studies cite injury severity and Medicaid insurance status as risk factors for transfer to level-I trauma centers from community hospitals.^17^, ^18^, ^19^ However, over the studied time period, the overall severity of hand injuries presenting to our institution remained the same. Additionally, unlike what is reported in previous studies, we noted an approximately 30% decrease in the percentage of Medicaid transfers and an 11% increase in the percentage of patients with commercial insurance when compared with the period before joining the Hand Trauma Center Network.

As previously stated, two major reasons why hand surgeons decline to take hand calls are lifestyle inconvenience and the disruption of elective call schedules.^11^ Knowing the approximate changes in the volume and severity of traumatic hand injuries may help hand surgeons and institutions to better prepare for absorbing the call burden with the least amount of disruption to each surgeon’s elective schedule and lifestyle. This can be performed through staffing accordingly and/or adjusting the income and the expected compensation of participating hand surgeons. Additionally, with no significant increase in the severity of traumatic hand injuries, surgeons should not expect significantly more complex cases.

## Limitations

Multiple limitations to our study exist. First, this is only a single-center retrospective analysis, and the values can differ based on the location of the trauma center in question. Additionally, we did not stratify our patients by the timing of surgery (urgent/emergent/overnight), specific upper-extremity severity score, or the number of replants/microvascular repairs. We also did not evaluate hospital revenue or physician productivity metrics before and after joining the registry, which may also affect the percentage of physicians interested in enrolling their trauma center into the Hand Trauma Center Network.

To conclude, with the high incidence of traumatic hand injuries and the many disparities in accessing care for such injuries, we hope to encourage further participation of institutions and providers in the ASSH Hand Trauma Center Network. Based on our findings, we believe new institutions and providers can expect anywhere from a 10% to a 60% increase in hand trauma burden without a significant change in the severity of the trauma cases after joining the network. Hopefully, as more trauma centers join the ASSH Hand Trauma Center Network, we can continue to close the gap in the accessibility and quality of care for traumatic hand injuries in the United States.
